# Computer-Aided Diagnosis of Equine Pharyngeal Lymphoid Hyperplasia Using the Object Detection-Based Processing Technique of Digital Endoscopic Images

**DOI:** 10.3390/ani15182758

**Published:** 2025-09-22

**Authors:** Natalia Kozłowska, Marta Borowska, Tomasz Jasiński, Małgorzata Wierzbicka, Małgorzata Domino

**Affiliations:** 1 Department of Large Animal Diseases and Clinic, Institute of Veterinary Medicine, Warsaw University of Life Sciences, 02-787 Warsaw, Poland; natalia_kozlowska@sggw.edu.pl (N.K.); tomasz_jasinski@sggw.edu.pl (T.J.); malgorzata_wierzbicka@sggw.edu.pl (M.W.); 2 Institute of Biomedical Engineering, Faculty of Mechanical Engineering, Białystok University of Technology, 15-351 Bialystok, Poland

**Keywords:** pharyngitis, lymphoid follicles, Voronoi diagram, image texture, horse

## Abstract

In human medicine, computer-aided diagnosis (CAD) is increasingly employed for screening, identifying, and monitoring early endoscopic signs of various diseases. However, its potential—despite proven benefits in human healthcare—remains largely underexplored in equine veterinary medicine. This study aimed to quantify endoscopic signs of pharyngeal lymphoid hyperplasia (PLH) as digital data and to assess their effectiveness in CAD of PLH in comparison and in combination with clinical data reflecting respiratory tract disease. Endoscopic images of the pharynx were collected from 70 horses clinically assessed as either healthy or affected by PLH. Digital data were extracted using an object detection-based processing technique and first-order statistics (FOS). The data were transformed using linear discriminant analysis (LDA) and classified with the random forest (RF) algorithm. Classification metrics were then calculated. When considering digital and clinical data, high classification performance was achieved (0.76 accuracy, 0.83 precision, 0.78 recall, and 0.76 F1 score), with the highest importance assigned to selected FOS features: Number of Objects and Neighbors, and Tracheal Auscultation. The proposed protocol of digitizing standard respiratory tract diagnostic methods provides effective discrimination of PLH grades, supporting the clinical value of CAD in veterinary medicine and paving the way for further research in digital medical diagnostics.

## Simple Summary

Artificial intelligence is increasingly being applied in medical practice, particularly in computer-aided diagnosis (CAD). While these applications are already common in humans, they have only recently been introduced in veterinary medicine, especially in equine practice. This study aimed to evaluate the effectiveness of CAD in diagnosing one of the respiratory tract diseases—pharyngeal lymphoid hyperplasia (PLH). Since PLH is visually diagnosed based on the size and number of lymphoid follicles within the pharyngeal mucosa, this study employed an object detection-based processing technique to identify lymphoid follicles on endoscopic images and combined it with two digitization approaches—Voronoi diagrams and first-order statistics (FOS)—to quantify endoscopic signs of PLH. A digital data set thus obtained from 70 horses was combined with a clinical data set, representing respiratory tract clinical symptoms, to assess classification performance using the machine learning algorithm. The proposed CAD method achieved the highest classification metrics—0.76 accuracy and 0.83 precision—when both data sets were combined. This performance was higher compared to applying the CAD method to either data set alone. The proposed CAD method provides effective discrimination of PLH grades and may be further applied to the assessment of equine pharyngeal endoscopic images.

## 1. Introduction

In recent years, artificial intelligence-based medical applications, such as computer-aided diagnosis (CAD) methods, have begun to support standard diagnostic practice by assisting clinicians and radiologists in analyzing medical images [1]. In human healthcare, CAD methods have been proposed for the automatic classification of, e.g., endoscopic images [1,2,3], ultrasound images [4], radiographic images [5], computed topographic (CT) images [6], magnetic resonance (MR) images [7], and positron emission tomography (PET) images in combination with CT [8] or MR [9]. CAD methods have been applied to screening, identifying, and monitoring early signs of diseases, providing important diagnostic benefits—such as reducing the risk of misdiagnosis, increasing diagnostic accuracy, and accelerating the diagnostic process—ultimately enabling earlier implementation of appropriate treatment [1,2]. In particular, many CAD methods have been developed for endoscopic imaging of the gastrointestinal tract, supporting the early diagnosis of polyps, ulcers, and perforations, thereby reducing both the incidence and mortality of gastrointestinal cancers [3,10,11,12,13]. In veterinary diagnostic imaging, especially in equine clinical practice and research, only a limited number of studies have addressed CAD applications. These include studies on the classification of radiographic images [14,15] and CT images [16], as well as the use of artificial intelligence to automate routine tasks such as CT image segmentation [17] and morphometric measurements [18,19]. However, to date, no research studies or case reports have investigated the application of CAD to endoscopic images in equine veterinary medicine, leaving this field largely underexplored. In this study, a procedure developed for human diagnostics was adapted for equine diagnostics to provide animals access to CAD and its benefits, as animals—like humans—also require a reduced risk of misdiagnosis, improved diagnostic accuracy, and a faster diagnostic process.

In equine veterinary medicine, pharyngitis—referred to as pharyngeal lymphoid hyperplasia (PLH)—is an upper respiratory tract disease diagnosed and graded visually by resting endoscopy [20,21]. The presence and severity of PLH are assessed on a 0–4 scale based on the endoscopic appearance of the pharyngeal mucosa [20,21]. Grade 0 represents a normal pharynx, whereas grades 1–4 indicate pharyngitis. Grades 1 and 2 are characterized by small lymphoid follicles located on the dorsal wall or dorsal and lateral walls of the pharynx, respectively. Grades 3 and 4 are characterized by large and edematous lymphoid follicles, respectively, regardless of location [20,21]. Since the number and size of lymphoid follicles increase with PLH severity, these follicles can be treated as objects, whose detection via object detection-based processing technique forms the basis for digitizing endoscopic signs. For PLH digitization, we propose quantifying both the proximity and area of detected objects/lymphoid follicles using Voronoi diagrams, as well as their texture using first-order statistics (FOS). Both approaches have been previously applied in equine research for digitizing thermographic images [22], radiographic images [23], and microscopic images [24].

Lymphoid follicles are composed of densely aggregated nodular lymphoid tissue within the pharyngeal mucosa and form part of the local immune system [25]. They represent the first line of immune defense against viral and bacterial infections [21,26], as well as against stabling-related irritant particles and allergens [27], playing a critical role in protecting the respiratory tract from infection and irritation-based inflammation [28,29]. When the local immune response to inhaled antigens and irritants becomes heightened, pharyngitis develops, and endoscopic signs of PLH become apparent [30]. In young horses (under 5 years old), PLH is considered a normal stage of upper airway immunological development, reflecting their initial exposure to environmental airborne stimuli [21,31]. In contrast, in older horses (over 5 years old), PLH occurs less frequently [32] and is associated with a more clinically significant active inflammatory process [21,33]. In such cases, PLH frequently co-occurs with other respiratory tract diseases, such as nasopharyngeal collapse [34], dorsal displacement of the soft palate [34,35,36], aryepiglottic fold collapse [34], guttural pouch infection [33], equine asthma [37], or influenza [38].

One may observe that early and accurate diagnosis of PLH addresses the needs of equine veterinarians, horse breeders, trainers, competitors, and, above all, horses. The proposed CAD protocol enables objective screening, identification, and monitoring of physiological data that are considered early signs of PLH, thereby improving our understanding of the clinical state of animals and allowing management, training, and treatment to be tailored to the needs of horses to maintain their welfare. For example, PLH in horses over 5 years old has been proposed as a predisposing factor for diseases of the upper airway—such as nasopharyngeal collapse [34], dorsal displacement of the soft palate [34,35,36], and aryepiglottic fold collapse [34]—as well as the lower airway, such as equine asthma [37], all of which significantly impair performance. This phenomenon arises because inflammation of the upper and lower airways typically shares the same underlying characteristics, as described in the concept of "unified airways", also referred to as “One Airway, One Disease” [39,40]. The anatomical continuity and histological similarity of airway segments, which underlie the functional integration of the upper and lower airways [40,41], make older horses with PLH more susceptible to co-occurring respiratory diseases. In these horses, faster and more accurate PLH diagnosis—encouraging equine veterinarians, horse breeders, trainers, and competitors to reduce training, limit exertion, and pursue more targeted diagnostics of the respiratory tract—will help avoid work by subclinical horses and accelerate recovery. 

By contrast, in horses under 5 years old, PLH is not considered a risk factor for poor performance [36,42,43,44], as it reflects the normal immunological development of the upper airway during this period [21,31]. In these horses, maintaining training despite PLH symptoms does not compromise welfare, as most young racehorses studied had no history of poor performance, even though approximately one-third of them presented grades 3–4 PLH [36,42,43]. However, even in young horses with PLH, further CAD and clinical monitoring may be beneficial by enabling earlier detection of potential health and welfare deterioration, as the following studies have indicated some relationships between physiological data and the needs and performance of young horses. One study reported that higher PLH grades were associated with a decreased likelihood of completing race [44]. Another study found that severe PLH was linked with impaired performance, evidenced by fewer starts, fewer wins, lower placing, and reduced earnings [45]. Another study demonstrated that 2- and 3-year-old horses with higher PLH grades showed reduced speed index, while 4-year-old and older horses with higher PLH grades exhibited impaired performance [46]. Given that all these large cohort studies focused on young thoroughbred racehorses [36,42,43,44,45,46], PLH in older horses appears to be underinvestigated.

This study aimed to quantify endoscopic signs of PLH as digital data and to evaluate their effectiveness in CAD of PLH, both in comparison with and in combination with clinical data reflecting respiratory tract diseases. Given that the clinical significance of PLH in young horses is debatable [36,42,43,44,45,46], this study was designed to enroll only horses older than 5 years old.

## 2. Materials and Methods

### 2.1. Study Design

This retrospective analytical study reviewed the clinical records of 219 warmblood horses that underwent resting endoscopy between January 2022 and January 2025 at the Equine Clinic of the Warsaw University of Life Sciences. The horses were privately owned patients, examined at their owners’ request due to abnormal respiratory noise, nasal discharge, coughing, or poor performance, or for pre-purchase evaluation.

The inclusion criteria were as follows: age over 5 years, a complete clinical record including clinical symptoms scores and endoscopic signs scores for PLH grading, endoscopy performed using the same flexible video bronchoscope, and availability of a high-quality endoscopic image of the pharynx. The exclusion criteria were as follows: age 5 years or younger, missing clinical symptoms scores, missing endoscopic signs scores for PLH grading, use of a different endoscope, absence of a saved endoscopic image of the pharynx, or poor image quality. Seventy clinical records (*n* = 70) met the inclusion criteria and were included in the study, while one hundred forty-eight clinical records (*n* = 149) were excluded, predominately due to too young age.

### 2.2. Clinical Data Collection

A detailed respiratory tract examination was performed according to the standard protocol [47]. This included scoring the following clinical symptoms of respiratory tract diseases: Respiratory Rate measurement (0–3 scores), evaluation of Nasal Discharge (0–2 scores), Tracheal Auscultation (0–3 scores), Thoracic Auscultation (0–3 scores), evaluation of Nasal Flare (0–1 scores), Cough Scoring (0–2 scores), and Abdominal Lift assessment (0–2 scores), using descriptors summarized in our previously published clinical symptoms scoring system [47] and outlined in Table 1. To ensure standardization of the clinical results, all horses were examined and then scored by the same two veterinarians (N.K. and M.W.). Any disagreements were resolved by a third party (M.D.).

Resting endoscopy was performed in accordance with international guidelines [48] using a flexible video bronchoscope (8 × 2000 mm; Karl Storz, Tuttlingen, Germany). The horses were sedated with detomidine hydrochloride (Domosedan; Orion Corporation, Espoo, Finland; 0.01 mg/kg body weight, i.v.) and butorphanol (Torbugesic; Zoetis Polska Sp. z o.o., Warsaw, Poland; 0.01 mg/kg body weight, i.v.), with doses calculated individually based on each horse’s body weight. During resting endoscopy, the nasopharynx, larynx, trachea, and tracheal septum were evaluated; however, only the pharyngeal region was analyzed in this study. When the endoscope was positioned in the pharynx anterior to the larynx, the digital images were frozen and saved as BMP files. At least five images of the pharynx were captured for each horse, while only one high-quality image—free of motion blur and clearly showing the pharyngeal vault, larynx, and the fully abducted epiglottis—was selected for further analysis. Additionally, the presence and amount of mucus on the dorsal and lateral walls of the pharynx were scored using our previously published scoring system (0–3 scores) [37].

Clinical data were then compiled as a series of 7 clinical symptoms and 1 Mucus Score for each horse and used for further analysis.

#### Clinical Grading of PLH

During resting endoscopy, the presence and grade of PLH [20,21] were assessed using an endoscopic signs scoring system for PLH grading [37], based on the descriptors outlined in Table 2. In this system, the pharyngeal mucosa was visually evaluated. The absence of visible lymphoid tissue was scored as 0, corresponding to grade 0 PLH. In contrast, the presence of lymphoid follicles was gradually scored from 1 to 4, where the presence of a few small lymphoid follicles was scored as 1, corresponding to grade 1 PLH, and the presence of numerous large, edematous lymphoid follicles was scored as 4, corresponding to grade 4 PLH. To ensure standardization of the endoscopic results, all horses were endoscopically examined and then scored by the same two veterinarians (N.K. and M.W.). Any disagreements were resolved by a third party (M.D.).

### 2.3. Digital Data Collection

**Figure 1:**
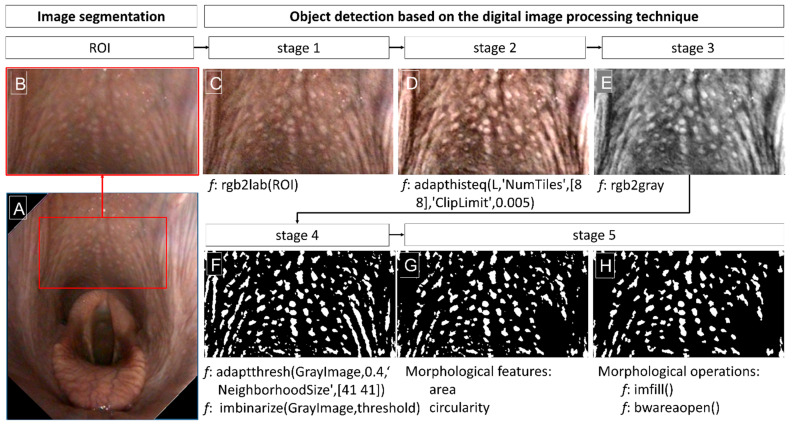
Endoscopic image processing workflow: a raw image after rotation and scaling (**A**), region of interest (ROI) after cropping from a raw image (**B**), image after preprocessing (stage 1) (**C**), image after contrast enhancement (stage 2) (**D**), image after conversion to grayscale (stage 3) (**E**), image after initial object detection (stage 4) (**F**), and image after refine object detection (stage 5) composed by removing too long objects based on the morphological features (**G**) and removing too small objects based on the morphological operations (**H**).

Each endoscopic image of the pharynx was initially rotated and scaled to a uniform larynx position and size, using a template consisting of a 46 mm × 58 mm oval positioned tangentially beneath a 50 mm × 90 mm rectangle. A raw image was scaled so that the oval aligned with the epiglottis in the vertical position, and the rectangle outlined the pharyngeal vault, ensuring a standardized surface area (Figure 1A). An image was then cropped to the dimensions of the rectangle, and a region of interest (ROI) measuring 297 pixels in height and 533 pixels in width was annotated and saved as a BMP file (Figure 1B).

ROIs were imported into MATLAB software version R2024b (MathWorks, Natick, Massachusetts, USA), and the object detection-based digital image processing technique was implemented in the following five key stages (1–5).

In stage 1—image preprocessing—each ROI was converted from the RGB color space to the CIE Lab color space. The conversion allows for independent manipulation of the lightness channel (L) without affecting the chromatic components. The component L was obtained from the MATLAB function rgb2lab(ROI), originally defined by Hunter [49] (Figure 1C).

In stage 2—contrast enhancement—adaptive histogram equalization, such as Contrast Limited Adaptive Histogram Equalization (CLAHE) algorithm, was applied to the lightness component to enhance local contrast in smaller regions of the image, while controlling noise amplification through contrast clipping. This operation was done using the MATLAB function adapthisteq(L,‘NumTiles’,[8 8],‘ClipLimit’,0.005) [50] (Figure 1D).

In stage 3—conversion to grayscale—ROIs were converted to grayscale using MATLAB function rgb2gray (Figure 1E).

In stage 4—initial object detection—adaptive thresholding was calculated using the MATLAB function adaptthresh(GrayImage,0.4,‘NeighborhoodSize’,[41 41]) and binarization was performed using the MATLAB function imbinarize(GrayImage, threshold). Adaptive thresholding was used to segment objects from the background based on local intensity variations, while binarization was used to convert the data into a binary format (Figure 1F).

In stage 5—refine object detection—morphological features and morphological operations were used to improve the detection of relevant objects. Morphological features, such as area and circularity, were applied to all detected objects. The morphological features were used to remove objects that were too long, such as folds located on the lateral wall of the pharynx and covering guttural pouch ostia (Figure 1G). Morphological operations, such as the MATLAB functions imfill() and bwareaopen(), were also applied to all detected objects. Function imfill() fills all holes in objects, and function bwareaopen deletes objects that are too small (Figure 1H).

Based on the described digital image processing technique, the objects representing the lymphoid follicles in the pharyngeal mucosa were detected, the Number of Objects was counted, and a Voronoi diagram was determined for each ROI represented by a set of objects *P* in a plane. The plane was divided into regions of influence—referred to as Voronoi regions—associated with points from the set *P*, such that each point in the associated region was closer to the associated object than to the other objects from the set *P* [51]. The shapes of the Voronoi regions depend on the distance metric, e.g., Euclidean distance, and the collection of all the Voronoi forms the Voronoi diagram. Voronoi diagrams were used to determine computational geometry by extracting the following Voronoi diagram features: Area, Area Over Voronoi, Number of Neighbors, Mean Neighbor Distance, Regularity, and Entropy [52,53,54].

Then, the texture features were extracted for each detected object using the MATLAB function regionprops() in MATLAB software version R2024b (MathWorks, Natick, MA, USA). The following features were extracted using formulas for FOS published in our previous study [22]: Mean, Standard Deviation, Median, Range, Variance, Skewness, Kurtosis, Root Mean Squared, Minimum, Maximum, 10th Percentile, 90th Percentile, Dominant 01, Dominant 10, Maximum of Moment 01, and Maximum of Moment 10.

For each detected object, 6 Voronoi diagram features and 16 FOS features were extracted. Given that the Number of Objects differed between PLH grades, the mean feature values were calculated for each image and used for further analysis.

### 2.4. Statistical Analysis

Clinical data (7 clinical symptoms, 1 Mucus Score) and digital data (6 Voronoi diagram features; 16 FOS features) were grouped for PLH grades and tested for Gaussian distributions using the Kolmogorov–Smirnov normality test. Each symptom/feature data series was compared between PLH grades using the ANOVA summary, when all data series were Gaussian distributed; or the Kruskal–Wallis test, when at least one data series was non-Gaussian distributed. The alpha value was established as α = 0.05. When significant differences were found in the first test, a post hoc test was performed. The ANOVA summary was followed by Holm–Sidak’s multiple comparisons test, while the Kruskal–Wallis test was followed by Dunn’s multiple comparisons test. Statistical analysis was performed using GraphPad Prism 6 software (GraphPad Software Inc., San Diego, CA, USA).

### 2.5. Endoscopic Image Classification

All data series were arranged into three data sets representing clinical data only, digital data only, and a combination of clinical and digital data (clinical+digital data). The same classification scheme and measurement of its effectiveness were used for each data set.

First, linear discriminant analysis (LDA) was used to reduce dimensionality [55]. The LDA approach simplifies classification [56] by transforming features from a high-dimensional space to a lower-dimensional space [57,58]. LDA was implemented using the scikit-learn library in Python software version 3.11 [59].

Then, the Random Forest (RF) algorithm was used to distinguish between five classes representing PLH grades 0–4. Features with the highest feature importance were used to train and evaluate a classification model [60,61]. Classification performance was evaluated using 5-fold stratified cross-validation accuracy. RF was implemented using the scikit-learn library in Python [59,62]. The classifier’s effectiveness was evaluated based on the following classification metrics: Accuracy, Precision, Recall, and F1.

## 3. Results

### 3.1. Clinical Data-Based Characteristics of PLH

Endoscopic signs of PLH grade 1 were observed in 16 horses (5 mares, 9 geldings, 2 stallions) with a median age of 16 years. PLH grade 2 was observed in 26 horses (12 mares, 14 geldings) with a median age of 12 years. PLH grade 3 was observed in 16 horses (6 mares, 10 geldings) with a median age of 10 years. PLH grade 4 was observed in 6 horses (5 mares, 1 geldings) with a median age of 9 years. No endoscopic signs of PLH (grade 0) were observed in 6 horses (1 mare, 2 geldings, 3 stallions) with a median age of 6 years. Horses with PLH grades 2–4 were younger than those with grade 0. Detailed demographic data are presented in Table 3.

**Figure 2:**
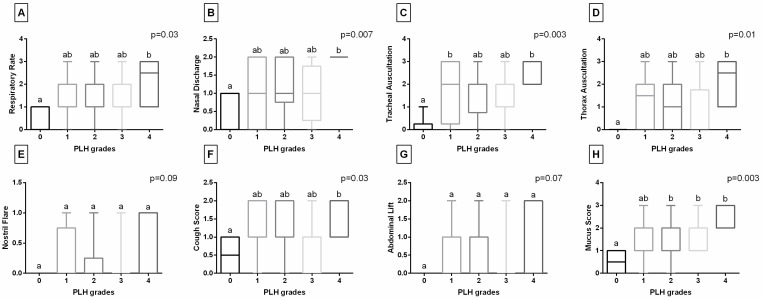
Comparison of clinical data - Respiratory Rate (**A**), Nasal Discharge (**B**), Tracheal Auscultation (**C**), Thorax Auscultation (**D**), Nostril Flare (**E**), Cough Score (**F**), Abdominal lift (**G**), and Mucus Score (**H**)—collected from horses with pharyngeal lymphoid hyperplasia (PLH) grades (0–4). Boxes represent median and lower and upper quartiles, while whiskers represent minimum and maximum values. Superscript letters indicate differences between PLH grades for *p* < 0.05.

Considering the clinical symptoms of respiratory tract diseases (Figure 2A–G), Respiratory Rate, Nasal Discharge, Thorax Auscultation, and Cough Score were higher in horses with PLH grade 4 compared to those with grade 0. Additionally, Tracheal Auscultation was higher in horses with PLH grades 1 and 4 than in those with grade 0. No differences were found between PLH grades for Nostril Flare and Abdominal Lift. When the clinical data set was complemented with endoscopic signs, Mucus Score was found to be higher in horses with PLH grades 2–4 compared to those with grade 0 (Figure 2H).

### 3.2. Digital Data-Based Characteristics of PLH

**Figure 3:**
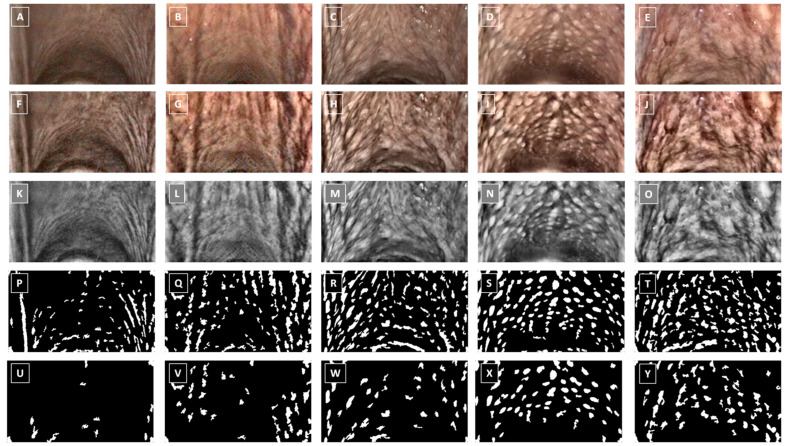
Endoscopic image of pharyngeal lymphoid hyperplasia (PLH) grade 0 (**A**,**F**,**K**,**P**,**U**), grade 1 (**B**,**G**,**L**,**Q**,**V**), grade 2 (**C**,**H**,**M**,**R**,**W**), grade 3 (**D**,**I**,**N**,**S**,**X**), and grade 4 (**E**,**J**,**O**,**T**,**Y**), represented by the raw images (**A**–**E**), images after contrast enhancement (**F**–**J**), images after conversion to grayscale (**K**–**O**), images after initial objects detection (**P**–**T**), and final images after with refine objects detection (**U**–**Y**).

The Number of Objects detected using the applied object detection-based processing technique was higher in horses with PLH grades 3–4 compared to those with grades 0–2 (Table 4), and their representations are shown in Figure 3.

**Figure 4:**
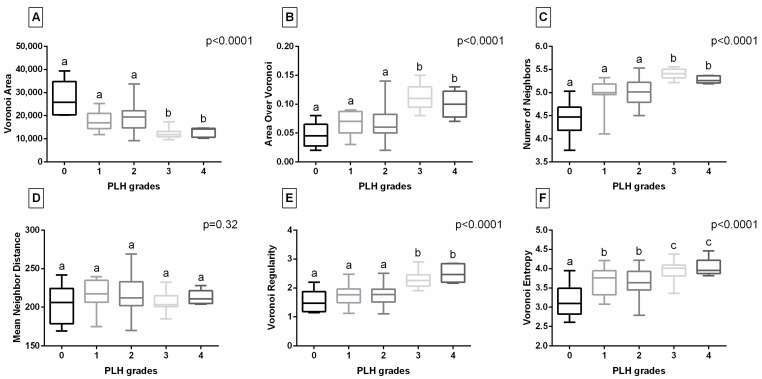
Comparison of Voronoi diagram features—Voronoi Area (**A**), Area Over Voronoi (**B**), Number of Neighbors (**C**), Mean Neighbor Distance (**D**), Voronoi Regulatiry (**E**), and Voronoi Entropy (**F**)—of endoscopic images representing pharyngeal lymphoid hyperplasia (PLH) grades (0–4). Boxes represent median and lower and upper quartiles, while whiskers represent minimum and maximum values. Superscript letters indicate differences between PLH grades for *p* < 0.05.

**Figure 5:**
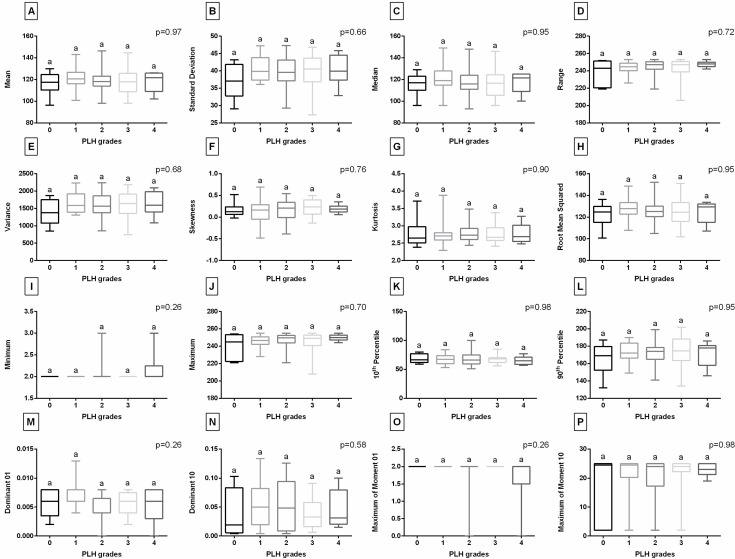
Comparison of the first-order statistics (FOS) features—Mean (**A**), Standard Deviation (**B**), Median (**C**), Range (**D**), Variance (**E**), Skewness (**F**), Kurtosis (**G**), Root Mean Squared (**H**), Minimum (**I**), Maximum (**J**), 10th Percentile (**K**), 90th Percentile (**L**), Dominant 01 (**M**), Dominant 10 (**N**), Maximum of Moment 01 (**O**), and Maximum of Moment 10 (**P**)—of endoscopic images representing pharyngeal lymphoid hyperplasia (PLH) grades (0–4). Boxes represent median and lower and upper quartiles, while whiskers represent minimum and maximum values. Superscript letters indicate differences between PLH grades for *p* < 0.05.

Considering the Voronoi diagram features of the endoscopic images studied (Figure 4), the Voronoi Area was lower in horses with PLH grades 3–4 compared to those with grades 0–2. The Area Over Voronoi, Number of Neighbors, and Voronoi Regularity were higher in horses with PLH grades 3–4 compared to those with grades 0–3. Additionally, Voronoi Entropy was higher in horses with PLH grades 3–4 than those with grades 0–3, as well as higher in horses with PLH grades 1–2 than those with grade 0. No differences were found between PLH grades for Voronoi Regularity. When the digital data set was complemented with the FOS features of the endoscopic images studied (Figure 5), no differences were found between PLH grades.

### 3.3. Computer-Aided Diagnosis of PLH

**Figure 6:**
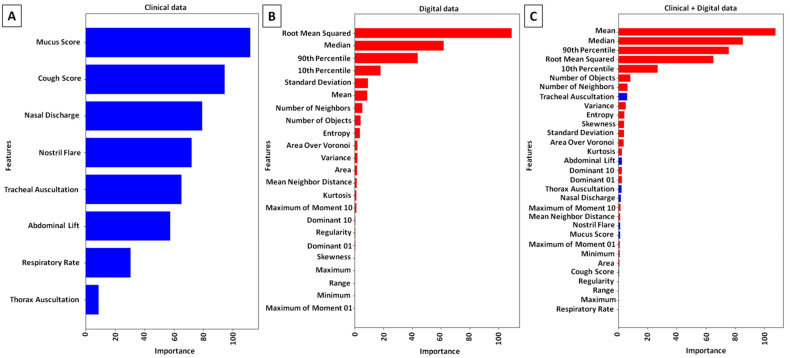
Feature importance of pharyngeal lymphoid hyperplasia (PLH) classification across three data sets: clinical data (**A**), digital data (**B**), as well as clinical and digital data (**C**). Clinical data are marked in blue, while digital data are marked in red.

When only the clinical data set was considered in PLH grades classification (Figure 6A), the following endoscopic signs and clinical symptoms had the highest importance for classification results: Mucus Score > Cough Score > Nasal Discharge > Nostril Flare > Tracheal Auscultation. However, this approach provides low classification metrics with 0.47 accuracy, 0.44 precision, 0.44 recall, and 0.41 F1 score (Table 5). When only the digital data set was considered in PLH grades classification (Figure 6B), the following FOS features and Voronoi diagram features had the highest importance for classification results: Root Mean Squared > Median > 90th Percentile > 10th Percentile > Standard Deviation > Mean > Number of Neighbors > Number of Objects. This approach provides higher classification metrics with 0.73 accuracy, 0.70 precision, 0.69 recall, and 0.65 F1 score (Table 5). When the clinical data set was combined with the digital data set for PLH grade classification (Figure 6C), the following FOS features, Voronoi diagram features, and clinical symptoms had the highest importance for the classification results: Mean > Median > 90th Percentile > Root Mean Squared > 10th Percentile > Number of Objects, Number of Neighbors > Tracheal Auscultations. This approach provides the highest classification metrics with 0.76 accuracy, 0.83 precision, 0.78 recall, and 0.76 F1 score (Table 5).

**Figure 7:**
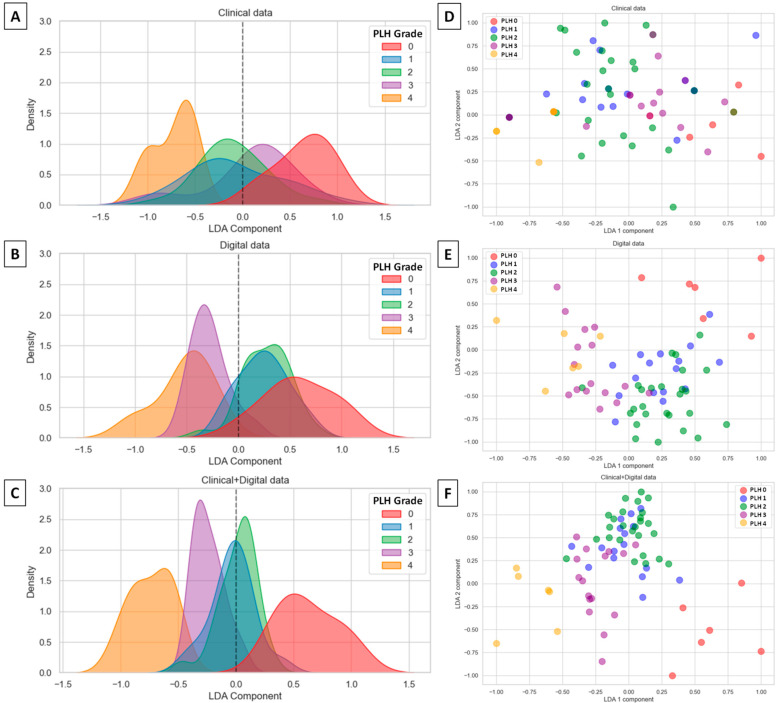
Distribution (**A**–**C**) and spatial distribution (**D**–**F**) of linear discriminant analysis (LDA) components of pharyngeal lymphoid hyperplasia (PLH) classification across three data sets: clinical data (**A**,**D**), digital data (**B**,**E**), as well as clinical and digital data (**C**,**F**).

One may observe that distribution of LDA components overlapped the most for the clinical data set, where the classes representing PLH grades 1–3 overlapped (Figure 7A). When the digital data set was considered, two overlaps were observed: PLH grade 3 with grade 4, and PLH grade 1 with grade 2 (Figure 7B). When the clinical data set and the digital data set were combined, only two classes representing PLH grades 1 and 2 overlapped (Figure 7C). The class overlaps were similarly visible in the spatial distribution scatter plots (Figure 7D–F), confirming the best separation between classes for the combined data set (Figure 7F).

## 4. Discussion

In this study, a horse-centered approach involves transferring and applying human CAD technology [1,2] to equine veterinary medicine, thereby developing and objectivizing PLH diagnosis and addressing the gap in our understanding of PLH in older horses. Among cohort studies on equine PLH, most have focused on young racehorses [36,42,43,44,45,46,63], with only two studies including horses older than 5 years [32,63]. Sweeney et al. reported PLH in 14% of horses over 5 years old [32], while Wichtel et al. observed PLH in 47% of horses aged 2 to 14 years [63]. Several studies on horses under 5 years old demonstrated that 2-year-old horses exhibited more severe PLH (grades 3–4) compared to 3-year-old and older horses [36,42,44,45]. Similarly, in Wichtel’s study on horses aged 2 to 14 years, those with higher PLH grades were younger than horses with grade 0 PLH [63]. Consistently, in this study on horses over 5 years old, we found that individuals with PLH grades 2, 3, and 4 were significantly younger than those with PLH grade 0. This aligns with the findings of Robinson et al., who reported that age is the only significant risk factor for PLH [64]. To date, no environmental risk factors have been identified for PLH prevalence. Clarke et al. found no association between poor stable ventilation and the presence or severity of PLH [43], and Auer et al. reported no significant differences in PLH severity among horses from different racetrack stables [42]. Therefore, the benefits of early diagnosis of PLH on broadly understood horses’ welfare have not yet been investigated.

Interestingly, only two analytic studies have reported the prevalence of PLH in adult horses that were referred to the equine clinic with respiratory tract diseases. In one study, PLH was observed in 8.2% of adult horses (mean ± SD age: 8.7 ± 2.6 years) [33], whereas in another study, PLH was reported in 90.0% of adult horses (mean ± SD age: 12.9 ± 4.6 years) [37]. Similarly, in this study, PLH was diagnosed in 91.4% of adult horses referred to our equine clinic with respiratory tract diseases. This large discrepancy in prevalence rates may be explained by methodological differences. In the first study [33], PLH and pharyngitis were counted separately, while in the second study [37], they were considered jointly. Furthermore, the latter study also included 4-year-old horses [37], which could have substantially overestimated PLH prevalence, given that Saulez et al. reported PLH in 63.1% of healthy 4-year-old horses [45].

One may observe that the CDA approach investigates clinical cases rather than subjecting research animals to invasive procedures for pharyngitis induction. On the one hand, experimentally induced disease is not always relevant, as it does not account for the anatomical continuity and histological similarity of airway segments [40,41], which may be associated with the development and progression of PLH [21,31], co-occurring respiratory diseases [34,35,36,37], and their clinical symptoms. On the other hand, incorporating clinical cases reduces the use of research animals, which is both beneficial for horses and ethically aligned with an animal-centered approach. Given that this study was conducted on horses exhibiting clinical symptoms of respiratory tract diseases, one may observe that the Mucus Score was higher in horses with PLH grades 2–4 compared to healthy horses. In most previous studies, the amount of tracheal mucus was assessed [43,44], whereas in this study, the amount of mucus on the pharyngeal wall was evaluated. However, our earlier study demonstrated that in horses with PLH, the amount of mucus in both the trachea and pharynx changes in a similar manner across PLH grades [37]. Therefore, the following discussion—although indirect—can be considered to reflect mucus accumulation in the upper airway in general. In one study, no association was found between the presence and severity of PLH and the amount of tracheal mucus [43]. In another study, the severity of PLH degree was associated with the amount of tracheal mucus, such that as PLH degree increased, tracheal Mucus Score increased [44]. Similarly, in our previous study, mucus accumulation in the upper airway—both in the pharynx and trachea—increased in parallel with PLH severity [37]. Such visible mucus accumulation in the trachea may serve as an indicator of inflammatory airway disease (IAD), although only when accompanied by an increased number of inflammatory cells within the mucus [65]. A limitation of this study is that inflammatory cells in the airway mucosa were not assessed. Nevertheless, Holcombe et al. reported an association between PLH grades and macrophage count in tracheal wash, with the number of cells increasing as PLH scores increased [44]. Furthermore, our earlier study showed 20–60% of neutrophils in tracheal wash from 18.3% of horses with PLH grades 1–3, and over 60% of neutrophils in tracheal wash from 58.8% of horses with PLH grades 1–3 [37]. Despite these findings, in the present study, the Mucus Score did not reach sufficient feature importance and was therefore not included in the RF classification model [60,61].

One may observe that the clinical symptoms of respiratory tract diseases were assessed in a standardized manner. To ensure consistency, a previously published clinical symptoms scoring system was applied [47], and the same team of veterinarians assessed the studied horses. This approach enabled comparison among the 70 horses and demonstrated that the Respiratory Rate, Nasal Discharge, and Cough Score were higher in horses with PLH grade 4 compared to healthy horses. Except for the Cough Score, no similar comparisons have been reported in previous studies. In one study, coughing was not considered a clinical symptom of PLH [44,66], while in another study reported a history of frequent coughing in horses with pharyngitis [33]. Another study highlighted the importance of including PLH in the differential diagnosis of cough, since horses with PLH grades 3–4 were often clinically diagnosed with cough [36]. This finding can be explained by the concept of “unified airways”, which emphasizes the anatomical continuity and functional integration of the respiratory tract [40,41]. Since cough is a reflex triggered by receptor stimulation anywhere along the airways—from the pharynx to the bronchi [67]—irritation of any airway segment can induce a cough. Despite these speculations, in the present study, the Respiratory Rate, Nasal Discharge, and Cough Score did not reach sufficient feature importance and were therefore not included in the RF classification model [60,61].

In only one previous study, abnormal respiratory noise was reported by the owner or trainer in horses with severe PLH. Interestingly, in those cases, PLH was the only respiratory disease detected [68]. Similarly, in this study, Tracheal Auscultation was higher in horses with PLH grades 1 and 4 compared to healthy horses, while Thorax Auscultation was higher in horses with PLH grade 4 compared to healthy horses. Kannegieter et al. suggested that the severity of PLH, along with potential co-occurring respiratory tract diseases, may contribute to respiratory insufficiency. Consequently, the authors recommended including PLH as a possible cause of respiratory insufficiency [68]. In line with this, our results supported the inclusion of one of the respiratory insufficiency symptoms in the PLH classifier. Interestingly, among all the evaluated clinical symptoms and endoscopic signs of respiratory tract diseases, only Tracheal Auscultation demonstrated high feature importance and was therefore incorporated into the RF classification model [60,61]. This combination of clinical data and digital data provided substantially higher classification performance (0.76 accuracy and 0.83 precision) than using either data set alone.

One may observe that when only clinical data were used for PLH classification, the RF algorithm incorporated Mucus Score, Cough Score, Nasal Discharge, and Nostril Flare in addition to Tracheal Auscultation. However, the resulting model demonstrated poor performance (0.43 accuracy and 0.44 precision), which was insufficient to consider CAD effective. By contrast, when only digital data were used, the RF algorithm incorporated six FOS features (Root Mean Squared, Median, both Percentiles, Standard Deviation, Mean) and two Voronoi diagram features (Number of Neighbors, Number of Objects), achieving higher—but still insufficient—classification performance (0.73 accuracy and 0.70 precision). Interestingly, similar FOS features (Mean, Median, both Percentiles, Root Mean Squared) and the same Voronoi diagram features (Number of Neighbors, Number of Objects) were incorporated—together with Tracheal Auscultation—into the best-performing classification model. Notably, the two most representative computational geometry features of lymphoid follicles—their number (Number of Objects) and the number of adjacent follicles (Number of Neighbors) [52,53,54]—demonstrated high feature importance across competing classification models. These findings suggest that the proposed rise of technologies—specifically, object detection-based processing technique—enables the detection and separation of objects representing the lymphoid follicles within the pharyngeal mucosa. This animal-centered technology may benefit horses by facilitating accelerated screening of a large equine cohort, thereby accurately identifying more horses with PLH, monitoring training of affected young horses, reducing exertion in affected older horses, enabling earlier implementation of appropriate treatment, tracking progression or remission of PLH, supporting more detailed diagnosis of concurrent airway diseases, and ultimately accelerating recovery while maintaining horse welfare.

Moreover, both the Number of Neighbors and the Number of Objects were higher in horses with PLH grades 3–4 compared to healthy horses and those with PLH grades 1–2. By contrast, none of the FOS features that achieved high feature importance in the RF model significantly differed between PLH grades. This may indicate that pharyngeal wall texture contributes to PLH differentiation; however, it is not sufficiently distinct for robust statistical discrimination. Therefore, further research into alternative image texture evaluation approaches is warranted. Given that the RF algorithm is an ensemble machine learning method that constructs multiple decision trees—each making predictions independently—with final classification determined by majority voting, it is well recognized for its high accuracy in multi-class classification tasks and robustness against overfitting [61,62]. These advantages motivated the RF algorithm selection for the current five-class classification (PLH grades 0–4) using a relatively small dataset of 70 realizations. However, no other supervised machine learning algorithms, such as k-nearest neighbors (kNN) [69,70] or support vector machine (SVM) [71,72], were investigated.

### Further Directions and Limitations

The lack of consideration of alternative image texture evaluation approaches, such as second-order statistics (SOS) [73,74], fractal dimension texture analysis [70], or entropy-based texture analysis, as well as other machine learning algorithms [69,70,71,72], represents both a limitation of this study and a direction for further research. Notably, SOS—such as Gray-Level Co-Occurrence Matrix (GLCM) [22,23], Gray-Level Run-Length Matrix (GRLM) [22,23], Gray Level Size Zone Matrix (GLSZM) [23], Gray Level Dependence Matrix (GLDM) [23], or Neighboring Gray Tone Difference Matrix (NGTDM) [23]—and entropy texture features [23,75] have already been successfully applied in previous equine research, suggesting that their application could also be effective in endoscopic image analysis. Moreover, findings from human studies indicate that while FOS/SOS alone may not always achieve optimal classification accuracy [73,74], they often complement other image digitization methods. Higher classification performance is typically obtained in mixed models combining large data sets of FOS, SOS, and other specific digitization methods, such as those based on entropy or fractals or Voronoi diagrams, as proposed in this study.

A second limitation of this study is the lack of consideration of other potentially important co-occurring diseases. One may observe that severe PLH is often indicative of active respiratory tract inflammation [21]. Moreover, this regional inflammation in the upper airway may predispose horses to other diseases of both the upper (nasopharyngeal collapse [34], dorsal displacement of the soft palate [34,35,36], aryepiglottic fold collapse [34]) and lower (equine asthma [37]) airways. For example, Kaiseler et al. demonstrated that horses with PLH grades 3–4 were more likely to develop dorsal displacement of the soft palate compared to those with PLH grades 1–2, suggesting a probable role of PLH in the etiopathogenesis of dorsal displacement of the soft palate [36]. Although this study included 70 horses, two of the five groups—those with the lowest and highest PLH grades—had relatively small sample sizes. Further stratification to account for the coexistence of the mentioned respiratory tract diseases would require a substantially larger cohort to ensure adequate subgroup representation. Since the primary aim of this study was to evaluate the effectiveness of CAD in PLH classification, only large-scale further studies that incorporate a balanced representation of co-occurring diseases will provide more comprehensive insight into the concept of “unified airways” [40,41].

## 5. Conclusions

Endoscopic signs of PLH may be quantified using Voronoi diagram features and FOS texture features, since the proposed object detection-based processing technique enables the detection and separation of objects representing the lymphoid follicles within the pharyngeal mucosa. In horses older than 5 years old, the proposed protocol of digitizing pharyngeal endoscopic images, combined with digitizing clinical symptoms of respiratory tract diseases, effectively discriminates PLH grades. Therefore, the proposed digitizing of these standard respiratory tract diagnostic methods may support the clinical value of CAD in veterinary medicine, paving the way for further research in digital medical diagnostics.

## Figures and Tables

**Table 1:** Clinical symptoms of respiratory tract diseases used for detailed respiratory tract examination scoring.

Clinical Symptoms	Descriptor	Score
Respiratory Rate	<16	0
	17–20	1
	21–30	2
	>30	3
Nasal Discharge	none	0
	serous	1
	mucopurulent/epistaxis	2
Tracheal Auscultation	normal tracheal sounds	0
	slight increase	1
	clearly audible increased	2
	crackles and wheezing present	3
Thorax Auscultation	normal pulmonary sounds	0
	slight increased pulmonary sounds	1
	clearly audible increased pulmonary sounds	2
	crackles and wheezing present	3
Nostril Flare	none	0
	present	1
Cough Score	none	0
	coughs at specific times of day (feeding/exercising/making beds)	1
	frequent cough	2
Abdominal Lift	none	0
	slight flattening of ventral flank	1
	obvious abdominal lift and “heave line”	2

**Table 2:** Endoscopic signs of the pharyngeal mucosa used for pharyngeal lymphoid hyperplasia (PLH) grading.

PLH Grade	Endoscopic Signs of the Pharyngeal Mucosa
Grade 0	No visible lymphoid tissue
Grade 1	A few small white lymphoid follicles on the dorsal wall of the pharynx
Grade 2	Numerous small lymphoid follicles on the dorsal and lateral walls of the pharynx
Grade 3	Numerous large hyperemic lymphoid follicles on the entire dorsal and lateral walls of the pharynx
Grade 4	Numerous large edematous hyperemic lymphoid follicles coalesce broad-based polypoid aggregate on the entire dorsal and lateral walls of the pharynx

**Table 3:** Demographic data (sex, age) of horses with pharyngeal lymphoid hyperplasia (PLH) grades (0–4). For sex, the number of mares, geldings, and stallions among the PLH groups is provided. The median and range (minimum and maximum values) are provided and compared for age. Superscript letters indicate differences in horses’ age between PLH grades for *p* < 0.05.

Demographic Data	PLH Grades				
	Grade 0	Grade 1	Grade 2	Grade 3	Grade 4
**Sex**					
Mare	1	5	12	6	5
Gelding	2	9	14	10	1
Stallion	3	2	0	0	0
In total	6	16	26	16	6
**Age**					
Median	16 ^a^ years	12 ^ab^ years	12 ^b^ years	10 ^b^ years	9 ^b^ years
Range (Min; Max)	(13; 19)	(6; 24)	(6; 18)	(6; 16)	(6; 14)
*p* value	*p* = 0.009				

**Table 4:** The Number of Objects representing lymphoid follicles, detected on the endoscopic images, and compared between pharyngeal lymphoid hyperplasia (PLH) grades (0–4). Median and range (minimum and maximum values) are provided. Superscript letters indicate differences between PLH grades for *p* < 0.05.

Number of Objects	PLH Grades				
	Grade 0	Grade 1	Grade 2	Grade 3	Grade 4
Median	18.0 ^a^	33.0 ^a^	30.5 ^a^	52.5 ^b^	50.5 ^b^
Range (Min; Max)	(12.0; 34.0)	(19.0; 44.0)	(15.0; 69.0)	(32.0; 75.0)	(37.0; 56.0)
*p* value	*p* < 0.0001				

**Table 5:** Classification metrics (accuracy, precision, recall, and F1 score) of pharyngeal lymphoid hyperplasia (PLH) classification across three data sets: clinical data, digital data, as well as clinical and digital data.

Classification Metrics	Clinical Data	Digital Data	Clinical + Digital Data
Accuracy	0.43	0.73	0.76
Precision	0.44	0.70	0.83
Recall	0.44	0.69	0.78
F1	0.41	0.65	0.76

## Data Availability

The data presented in this study are available on request from the corresponding author.

## References

[1] Mukhtorov D., Rakhmonova M., Muksimova S., Cho Y.-I. (2023). Endoscopic Image Classification Based on Explainable Deep Learning. Sensors.

[2] Ahmad J., Muhammad K., Lee M.Y., Baik S.W. (2017). Endoscopic Image Classification and Retrieval Using Clustered Convolutional Features. J. Med. Syst..

[3] Yang X., Wei Q., Zhang C., Zhou K., Kong L., Jiang W. (2020). Colon Polyp Detection and Segmentation Based on Improved MRCNN. IEEE Trans. Instrum. Meas..

[4] Ragab M., Albukhari A., Alyami J., Mansour R.F. (2022). Ensemble Deep-Learning-Enabled Clinical Decision Support System for Breast Cancer Diagnosis and Classification on Ultrasound Images. Biology.

[5] Ashwini S., Arunkumar J., Prabu T., Singh N., Singh N. (2024). Diagnosis and Multi-Classification of Lung Diseases in CXR Images Using Optimized Deep Convolutional Neural Network. Soft Comput..

[6] Ozsahin I., Sekeroglu B., Musa M.S., Mustapha M.T., Uzun Ozsahin D. (2020). Review on Diagnosis of COVID-19 from Chest CT Images Using Artificial Intelligence. Comput. Math. Methods Med..

[7] Cheng D., Liu M., Fu J., Wang Y. Classification of MR brain images by combination of multi-CNNs for AD diagnosis. Proceedings of the 9th International Conference on Digital Image Processing (ICDIP 2017).

[8] Han Y., Ma Y., Wu Z., Zhang F., Zheng D., Liu X., Tao L., Liang Z., Yang Z., Li X. (2021). Histologic Subtype Classification of Non-Small Cell Lung Cancer Using PET/CT Images. Eur. J. Nucl. Med. Mol. Imaging.

[9] Kebir S., Weber M., Lazaridis L., Deuschl C., Schmidt T., Mönninghoff C., Keyvani K., Umutlu L., Pierscianek D., Forsting M. (2019). Hybrid 11C-MET PET/MRI Combined with “Machine Learning” in Glioma Diagnosis According to the Revised Glioma WHO Classification 2016. Clin. Nucl. Med..

[10] Banik D., Roy K., Bhattacharjee D., Nasipuri M., Krejcar O. (2020). Polyp-Net: A Multi-Model Fusion Network for Polyp Segmentation. IEEE Trans. Instrum. Meas..

[11] Gaur P., Gupta H., Chowdhury A., Mc Creadie K., Pachori R.B., Wang H. (2021). A Sliding Window Common Spatial Pattern for Enhancing Motor Imagery Classification in EEG-BCI. IEEE Trans. Instrum. Meas..

[12] Yue G., Han W., Jiang B., Zhou T., Cong R., Wang T. (2022). Boundary Constraint Network with Cross Layer Feature Integration for Polyp Segmentation. IEEE J. Biomed. Health Inform..

[13] Yue G., Wei P., Liu Y., Luo Y., Du J., Wang T. (2023). Automated Endoscopic Image Classification via Deep Neural Network with Class Imbalance Loss. IEEE Trans. Instrum. Meas..

[14] Guo L., Yu X., Thair A., Rideout A., Collins A., Wang Z.J., Hore M. (2024). Deep Learning Model Shows Promise for Detecting and Grading Sesamoiditis in Horse Radiographs. Am. J. Vet. Res..

[15] Turek B., Pawlikowski M., Jankowski K., Borowska M., Skierbiszewska K., Jasiński T., Domino M. (2025). Selection of Density Standard and X-Ray Tube Settings for Computed Digital Absorptiometry in Horses Using the k-Means Clustering Algorithm. BMC Vet. Res..

[16] Borowska M. (2023). Wprowadzenie do Zastosowania Entropii w Analizie Sygnałów i Obrazów Biomedycznych Oraz Jej Aplikacje w Medycynie i Weterynarii.

[17] Aszteborski H., Koneck K., Jasiński T., Borowska M., Lipowicz P., Turek B., Domino M. AI-Driven Automatic 3D Segmentation of Equine Maxillary Sinuses—Integrating Convolutional Neural Networks in Veterinary Diagnostic Imaging. Proceedings of the European Veterinary Diagnostic Imaging Conference (EVDI).

[18] Bakıcı C., Batur B., Akgün R., Kaya U., Ekim O., Oto Ç., Hazıroğlu R. (2022). Computer-Aided Three Dimensional Morphometric Measurements of Cervical Vertebrae Variations Compared with Manual Measurements in Throughbred Horses. Eurasian J. Vet. Sci..

[19] Borowska M., Lipowicz P., Daunoravičienė K., Turek B., Jasiński T., Pauk J., Domino M. (2024). Three-Dimensional Segmentation of Equine Paranasal Sinuses in Multidetector Computed Tomography Datasets: Preliminary Morphometric Assessment Assisted with Clustering Analysis. Sensors.

[20] Raker C.W., Boles C.L. (1978). Pharyngeal Lymphoid Hyperplasia in the Horse. J. Equine Med. Surg..

[21] Holcombe S., Ducharme N., McGorum B., Dixon P., Robinson E., Schumacher J. (2007). Disorders of the Nasopharynx and Soft Palate. Equine Respiratory Medicine and Surgery.

[22] Domino M., Borowska M., Kozłowska N., Trojakowska A., Zdrojkowski Ł., Jasiński T., Smyth G., Maśko M. (2022). Selection of Image Texture Analysis and Color Model in the Advanced Image Processing of Thermal Images of Horses Following Exercise. Animals.

[23] Górski K., Borowska M., Stefanik E., Polkowska I., Turek B., Bereznowski A., Domino M. (2022). Selection of Filtering and Image Texture Analysis in the Radiographic Images Processing of Horses’ Incisor Teeth Affected by the EOTRH Syndrome. Sensors.

[24] Toutain M., Lezoray O., Audigié F., Busoni V., Rossi G., Parillo F., Elmoataz A. Analysis of Whole Slide Images of Equine Tendinopathy. Proceedings of the ICIAR (International Conference on Image Analysis and Recognition).

[25] Mair T.S., Batten E.H., Stokes C.R., Bourne F.J. (1987). The Histological Features of the Immune System of the Equine Respiratory Tract. J. Comp. Pathol..

[26] McAllister E.S., Blakeslee J.R. (1977). Clinical Observations of Pharyngitis in the Horse. J. Am. Vet. Med. Assoc..

[27] Holcombe S.J., Jackson C., Gerber V., Jefcoat A., Berney C., Eberhardt S., Robinson N.E. (2001). Stabling Is Associated with Airway Inflammation in Young Arabian Horses. Equine Vet. J..

[28] Galan J.E., Timoney J.F. (1985). Mucosal Nasopharyngeal Immune Responses of Horses to Protein Antigens of *Streptococcus equi*. Infect. Immun..

[29] Breathnach C.C., Yeargan M.R., Sheoran A.S., Allen G.P. (2001). The Mucosal Humoral Immune Response of the Horse to Infective Challenge and Vaccination with Equine Herpesvirus-1 Antigens. Equine Vet. J..

[30] Ahern T.J. (1997). Tonsillitis and Tonsillar Hypertrophy Predisposing to Pharyngeal Dysfunction in the Horse. J. Equine Vet. Sci..

[31] Holcombe S.J. (2005). Epidemiology of Airway Inflammation and Mucus in Horses.

[32] Sweeney C.R., Maxson A.D., Soma L.R. (1991). Endoscopic Findings in the Upper Respiratory Tract of 678 Thoroughbred Racehorses. J. Am. Vet. Med. Assoc..

[33] Gajarlwar O.S., Suryawanshi R.V., Ulemale A.H., Rangnekar M.N., Khambatta P. (2020). Prevalence of Upper Respiratory Tract Diseases in Thoroughbred Racehorses. Haryana Vet..

[34] King D.S., Tulleners E., Martin B.B., Parente E.J., Boston R. (2001). Clinical Experiences with Axial Deviation of the Aryepiglottic Folds in 52 Racehorses. Vet. Surg..

[35] Courouce-Malblanc A., Deniau V., Rossignol F., Corde R., Leleu C., Maillard K., Pitel P.-H., Pronost S., Fortier G. (2010). Physiological Measurements and Prevalence of Lower Airway Diseases in Trotters with Dorsal Displacement of the Soft Palate. Equine Vet. J..

[36] Kaiseler P.H., Dzyekanski B., Schiefelbein R., Silveira R.G., Pimpão C.T., Michelotto P.V. (2012). Upper Airway Evaluations of Thoroughbred Racehorses in a Private Clinic in Curitiba, Brazil—Resitng Endoscopic Findings in 587 Horses. Arch. Vet. Sci..

[37] Kozłowska N., Wierzbicka M., Jasiński T., Domino M. (2024). Co-Occurrence of Equine Asthma and Pharyngeal Lymphoid Hyperplasia in Pleasure Horses. Agriculture.

[38] Wilkins P.A. (2003). Lower Airway Diseases of the Adult Horse. Vet. Clin. Equine Pract..

[39] Grossman J. (1997). One Airway, One Disease. Chest.

[40] Giovannini-Chami L., Paquet A., Sanfiorenzo C., Pons N., Cazareth J., Magnone V., Lebrigand K., Chevalier B., Vallauri A., Julia V. (2018). The “One Airway, One Disease” Concept in Light of Th2 Inflammation. Eur. Respir. J..

[41] Holcombe S., Hinchcliff K.W., Geor R.J., Kaneps A.J. (2008). Upper Airway Function of Normal Horses during Exercise. Equine Exercise Physiology: The Science of Exercise in the Athletic Horse.

[42] Auer D.E., Wilson R.G., Groenendyk S. (1985). Pharyngeal Lymphoid Hyperplasia in Thoroughbred Racehorses in Training. Aust. Vet. J..

[43] Clarke A.F., Madelin T.M., Allpress R.G. (1987). The Relationship of Air Hygiene in Stables to Lower Airway Disease and Pharyngeal Lymphoid Hyperplasia in Two Groups of Thoroughbred Horses. Equine Vet. J..

[44] Holcombe S.J., Robinson N.E., Derksen F.J., Bertold B., Genovese R., Miller R., de Feiter Rupp H., Carr E.A., Eberhart S.W., Boruta D. (2006). Effect of Tracheal Mucus and Tracheal Cytology on Racing Performance in Thoroughbred Racehorses. Equine Vet. J..

[45] Saulez M.N., Gummow B. (2009). Prevalence of Pharyngeal, Laryngeal and Tracheal Disorders in Thoroughbred Racehorses, and Effect on Performance. Vet. Rec..

[46] Bagshaw J., Sanz M., Wang Y., Shoemaker S., Bayly W. (2023). Severity and Effects of Pharyngeal Lymphoid Hyperplasia Vary with Age and Racetrack Location in Thoroughbred Racehorses. Comp. Exerc. Physiol..

[47] Kozłowska N., Wierzbicka M., Pawliński B., Domino M. (2023). Co-Occurrence of Severe Equine Asthma and Palatal Disorders in Privately Owned Pleasure Horses. Animals.

[48] Fjeldborg J., Lindegaard C., van Erck-Westergren E., Strand E., Hackett E. (2024). Endoscopic Examination of the Respiratory Tract. Equine Respiratory Endoscopy.

[49] Hunter R.S. (1958). Photoelectric Color Difference Meter. J. Opt. Soc. Am..

[50] Zuiderveld K. (1994). Contrast Limited Adaptive Histogram Equalization. Graphics Gems IV.

[51] Dobrin A. (2005). A Review of Properties and Variations of Voroni Diagrams.

[52] Huang Z., Huang G., Cheng L. (2018). Medical Image Segmentation of Blood Vessels Based on Clifford Algebra and Voronoi Diagram. J. Softw..

[53] Sudbø J., Marcelpoil R., Reith A. (2000). New Algorithms Based on the Voronoi Diagram Applied in a Pilot Study on Normal Mucosa and Carcinomas. Anal. Cell. Pathol..

[54] Lau C., Kalantari B., Batts K.P., Ferrell L.D., Nyberg S.L., Graham R.P., Moreira R.K. (2021). The Voronoi Theory of the Normal Liver Lobular Architecture and Its Applicability in Hepatic Zonation. Sci. Rep..

[55] Lin W., Gao Q., Du M., Chen W., Tong T. (2021). Multiclass Diagnosis of Stages of Alzheimer’s Disease Using Linear Discriminant Analysis Scoring for Multimodal Data. Comput. Biol. Med..

[56] Adebiyi M.O., Arowolo M.O., Mshelia M.D., Olugbara O.O. (2022). A Linear Discriminant Analysis and Classification Model for Breast Cancer Diagnosis. Appl. Sci..

[57] Rezaei Z. (2021). A Review on Image-Based Approaches for Breast Cancer Detection, Segmentation, and Classification. Expert Syst. Appl..

[58] Bechelli S., Delhommelle J. (2022). Machine Learning and Deep Learning Algorithms for Skin Cancer Classification from Dermoscopic Images. Bioengineering.

[59] Pedregosa F., Varoquaux G., Gramfort A., Michel V., Thirion B., Grisel O., Blondel M., Prettenhofer P., Weiss R., Dubourg V. (2011). Scikit-Learn: Machine Learning in Python. J. Mach. Learn. Res..

[60] Babenko V., Nastenko I., Pavlov V., Horodetska O., Dykan I., Tarasyuk B., Lazoryshinets V. (2023). Classification of Pathologies on Medical Images Using the Algorithm of Random Forest of Optimal-Complexity Trees. Cybern. Syst. Anal..

[61] Jasti V.D.P., Zamani A.S., Arumugam K., Naved M., Pallathadka H., Sammy F., Raghuvanshi A., Kaliyaperumal K. (2022). Computational Technique Based on Machine Learning and Image Processing for Medical Image Analysis of Breast Cancer Diagnosis. Secur. Commun. Netw..

[62] Breiman L. (2001). Random Forests. Mach. Learn..

[63] Wichtel M., Gomez D., Burton S., Wichtel J., Hoffman A. (2016). Relationships between Equine Airway Reactivity Measured by Flowmetric Plethysmography and Specific Indicators of Airway Inflammation in Horses with Suspected Inflammatory Airway Disease. Equine Vet. J..

[64] Robinson N.E., Berney C., Behan A., Derksen F.J. (2009). Fluticasone Propionate Aerosol Is More Effective for Prevention than Treatment of Recurrent Airway Obstruction. J. Vet. Intern. Med..

[65] Couetil L.L., Rosenthal F.S., DeNicola D.B., Chilcoat C.D. (2001). Clinical Signs, Evaluation of Bronchoalveolar Lavage Fluid, and Assessment of Pulmonary Function in Horses with Inflammatory Respiratory Disease. Am. J. Vet. Res..

[66] Robinson N.E., Karmaus W., Holcombe S.J., Carr E.A., Derksen F.J. (2006). Airway Inflammation in Michigan Pleasure Horses: Prevalence and Risk Factors. Equine Vet. J..

[67] Couëtil L.L., Hoffman A.M., Hodgson J., Buechner-Maxwell V., Viel L., Wood J.L.N., Lavoie J.-P. (2007). Inflammatory Airway Disease of Horses. J. Vet. Intern. Med..

[68] Kannegieter N.J., Dore M.L. (1995). Endoscopy of the Upper Respiratory Tract during Treadmill Exercise: A Clinical Study of 100 Horses. Aust. Vet. J..

[69] Xing X., Jia X., Meng M.-H.Q. (2019). Bleeding Detection in Wireless Capsule Endoscopy Image Video Using Superpixel-Color Histogram and a Subspace KNN Classifier. Proceedings of the 1st International Conference on Advances in Science, Engineering and Robotics Technology 2019.

[70] Bębas E., Pauk K., Pauk J., Daunoravičienė K., Mojsak M., Hładuński M., Domino M., Borowska M. (2025). Application of Fractal Radiomics and Machine Learning for Differentiation of Non-Small Cell Lung Cancer Subtypes on PET/MR Images. J. Clin. Med..

[71] Li B., Meng M.Q.-H. (2012). Tumor Recognition in Wireless Capsule Endoscopy Images Using Textural Features and SVM-Based Feature Selection. IEEE Trans. Inf. Technol. Biomed..

[72] Bębas E., Borowska M., Derlatka M., Oczeretko E., Hładuński M., Szumowski P., Mojsak M. (2021). Machine-Learning-Based Classification of the Histological Subtype of Non-Small-Cell Lung Cancer Using MRI Texture Analysis. Biomed. Signal Process. Control.

[73] Hossain M.R.I., Ahmed I., Kabir M., Jawahar C.V., Shan S. (2015). Automatic Lung Tumor Detection Based on GLCM Features. Computer Vision, Proceedings of the ACCV 2014 Workshops, Singapore, 1–2 November 2014.

[74] Adi K., Widodo C.E., Widodo A.P., Gernowo R., Pamungkas A., Syifa R.A. (2018). Detection Lung Cancer Using Gray Level Co-Occurrence Matrix (GLCM) and Back Propagation Neural Network Classification. J. Eng. Sci. Technol. Rev..

[75] Domino M., Borowska M., Zdrojkowski Ł., Jasiński T., Sikorska U., Skibniewski M., Maśko M. (2022). Application of the Two-Dimensional Entropy Measures in the Infrared Thermography-Based Detection of Rider: Horse Bodyweight Ratio in Horseback Riding. Sensors.

